# Supplementary material for: Active and water-soluble form of lipidated Wnt protein is maintained by a serum glycoprotein afamin/α-albumin
Source: eLife. 2016 Feb 23;5:e11621. doi: 10.7554/eLife.11621 (PMC4775226; doi:10.7554/eLife.11621)
Supplement: Supplementary file 1. — Amino acid sequence of the N-terminal regions for the all tagged Wnt protein constructs used in this study starting at the initiation Met are shown. DOI: http://dx.doi.org/10.7554/eLife.11621.024 [file elife-11621-supp1.pptx]

## Slide 1
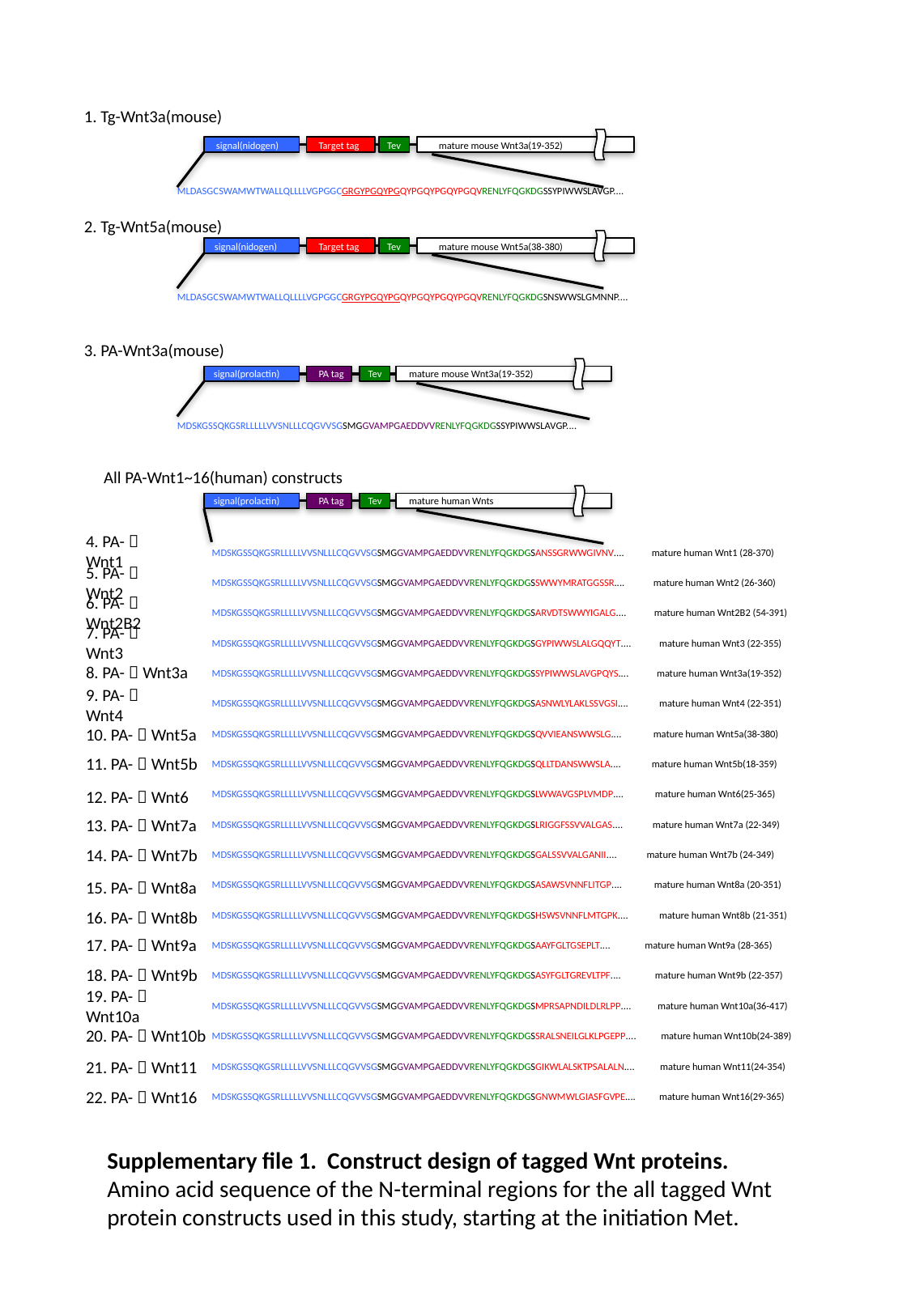

1. Tg-Wnt3a(mouse)
signal(nidogen)
Target tag
Tev
 mature mouse Wnt3a(19-352)
MLDASGCSWAMWTWALLQLLLLVGPGGCGRGYPGQYPGQYPGQYPGQYPGQVRENLYFQGKDGSSYPIWWSLAVGP....
2. Tg-Wnt5a(mouse)
signal(nidogen)
Target tag
Tev
 mature mouse Wnt5a(38-380)
MLDASGCSWAMWTWALLQLLLLVGPGGCGRGYPGQYPGQYPGQYPGQYPGQVRENLYFQGKDGSNSWWSLGMNNP....
3. PA-Wnt3a(mouse)
signal(prolactin)
PA tag
Tev
 mature mouse Wnt3a(19-352)
MDSKGSSQKGSRLLLLLVVSNLLLCQGVVSGSMGGVAMPGAEDDVVRENLYFQGKDGSSYPIWWSLAVGP....
All PA-Wnt1~16(human) constructs
signal(prolactin)
PA tag
Tev
 mature human Wnts
4. PA-ｈWnt1
MDSKGSSQKGSRLLLLLVVSNLLLCQGVVSGSMGGVAMPGAEDDVVRENLYFQGKDGSANSSGRWWGIVNV....
 mature human Wnt1 (28-370)
5. PA-ｈWnt2
MDSKGSSQKGSRLLLLLVVSNLLLCQGVVSGSMGGVAMPGAEDDVVRENLYFQGKDGSSWWYMRATGGSSR....
 mature human Wnt2 (26-360)
6. PA-ｈWnt2B2
MDSKGSSQKGSRLLLLLVVSNLLLCQGVVSGSMGGVAMPGAEDDVVRENLYFQGKDGSARVDTSWWYIGALG....
 mature human Wnt2B2 (54-391)
7. PA-ｈWnt3
MDSKGSSQKGSRLLLLLVVSNLLLCQGVVSGSMGGVAMPGAEDDVVRENLYFQGKDGSGYPIWWSLALGQQYT....
 mature human Wnt3 (22-355)
8. PA-ｈWnt3a
MDSKGSSQKGSRLLLLLVVSNLLLCQGVVSGSMGGVAMPGAEDDVVRENLYFQGKDGSSYPIWWSLAVGPQYS....
 mature human Wnt3a(19-352)
9. PA-ｈWnt4
MDSKGSSQKGSRLLLLLVVSNLLLCQGVVSGSMGGVAMPGAEDDVVRENLYFQGKDGSASNWLYLAKLSSVGSI....
 mature human Wnt4 (22-351)
10. PA-ｈWnt5a
MDSKGSSQKGSRLLLLLVVSNLLLCQGVVSGSMGGVAMPGAEDDVVRENLYFQGKDGSQVVIEANSWWSLG....
 mature human Wnt5a(38-380)
11. PA-ｈWnt5b
MDSKGSSQKGSRLLLLLVVSNLLLCQGVVSGSMGGVAMPGAEDDVVRENLYFQGKDGSQLLTDANSWWSLA....
 mature human Wnt5b(18-359)
MDSKGSSQKGSRLLLLLVVSNLLLCQGVVSGSMGGVAMPGAEDDVVRENLYFQGKDGSLWWAVGSPLVMDP....
 mature human Wnt6(25-365)
12. PA-ｈWnt6
13. PA-ｈWnt7a
MDSKGSSQKGSRLLLLLVVSNLLLCQGVVSGSMGGVAMPGAEDDVVRENLYFQGKDGSLRIGGFSSVVALGAS....
 mature human Wnt7a (22-349)
14. PA-ｈWnt7b
MDSKGSSQKGSRLLLLLVVSNLLLCQGVVSGSMGGVAMPGAEDDVVRENLYFQGKDGSGALSSVVALGANII....
 mature human Wnt7b (24-349)
MDSKGSSQKGSRLLLLLVVSNLLLCQGVVSGSMGGVAMPGAEDDVVRENLYFQGKDGSASAWSVNNFLITGP....
 mature human Wnt8a (20-351)
15. PA-ｈWnt8a
16. PA-ｈWnt8b
MDSKGSSQKGSRLLLLLVVSNLLLCQGVVSGSMGGVAMPGAEDDVVRENLYFQGKDGSHSWSVNNFLMTGPK....
 mature human Wnt8b (21-351)
17. PA-ｈWnt9a
MDSKGSSQKGSRLLLLLVVSNLLLCQGVVSGSMGGVAMPGAEDDVVRENLYFQGKDGSAAYFGLTGSEPLT....
 mature human Wnt9a (28-365)
18. PA-ｈWnt9b
MDSKGSSQKGSRLLLLLVVSNLLLCQGVVSGSMGGVAMPGAEDDVVRENLYFQGKDGSASYFGLTGREVLTPF....
 mature human Wnt9b (22-357)
19. PA-ｈWnt10a
MDSKGSSQKGSRLLLLLVVSNLLLCQGVVSGSMGGVAMPGAEDDVVRENLYFQGKDGSMPRSAPNDILDLRLPP....
 mature human Wnt10a(36-417)
20. PA-ｈWnt10b
MDSKGSSQKGSRLLLLLVVSNLLLCQGVVSGSMGGVAMPGAEDDVVRENLYFQGKDGSSRALSNEILGLKLPGEPP....
 mature human Wnt10b(24-389)
21. PA-ｈWnt11
MDSKGSSQKGSRLLLLLVVSNLLLCQGVVSGSMGGVAMPGAEDDVVRENLYFQGKDGSGIKWLALSKTPSALALN....
 mature human Wnt11(24-354)
22. PA-ｈWnt16
MDSKGSSQKGSRLLLLLVVSNLLLCQGVVSGSMGGVAMPGAEDDVVRENLYFQGKDGSGNWMWLGIASFGVPE....
 mature human Wnt16(29-365)
Supplementary file 1. Construct design of tagged Wnt proteins.
Amino acid sequence of the N-terminal regions for the all tagged Wnt protein constructs used in this study, starting at the initiation Met.
